# Utility of Reflex CMV Immunohistochemistry in Patients with Inflammatory Bowel Disease

**DOI:** 10.5146/tjpath.2025.14375

**Published:** 2025-09-30

**Authors:** Melek Buyuk, Neslihan Berker, Dogu Vuralli Bakkaloglu, Elif Eroglu, Sevim Mese, Bilger Cavus, Aslı Cifcibasi Ormeci, Mustafa Onel, Ali Agacfidan, Mine Gulluoglu

**Affiliations:** Department of Pathology, Istanbul University Istanbul Faculty of Medicine, Istanbul, Türkiye; Department of Medical Microbiology, Istanbul University Istanbul Faculty of Medicine, Istanbul, Türkiye; Department of Gastroenterology and Hepatology, Istanbul University Istanbul Faculty of Medicine, Istanbul, Türkiye

**Keywords:** Inflammatory bowel diseases, CMV, Immunohistochemistry, Tissue PCR, Endoscopic biopsy

## Abstract

***Objective: *** We aimed to investigate the association between CMV immunohistochemistry positivity and clinical, endoscopic, histologic, and tissue CMV PCR findings in ileocolonoscopic biopsies of inflammatory bowel disease patients, and to assess the diagnostic value of CMV immunohistochemistry as a reflex test during routine histopathologic evaluation.

***Material and Methods:*** We conducted a retrospective analysis of 191 patients (136 ulcerative colitis, 55 Crohn’s disease) between 2018 and 2021. We analyzed clinical data, endoscopic Mayo scores, histologic activity (Simplified Geboes Score), cytopathic changes, CMV immunohistochemistry and tissue CMV PCR results.

***Results: *** CMV immunohistochemistry was positive in 32.4% of cases, significantly associated with ulcerative colitis (p=0.003), symptomatic presentation (p=0.001), extensive colonic involvement (p<0.001), high histologic activity scores (p<0.001), and ulceration (p<0.001). Notably, 74.2% of CMV immunohistochemistry-positive cases had no preliminary clinical suspicion of CMV infection. Viral cytopathic changes were identified in only 30.6% of immunopositive cases on hematoxylin-eosin staining. CMV immunohistochemistry showed a significant correlation with tissue PCR (p<0.001), although some discordant cases occurred. The PCR-positive group had significantly higher immunopositive cell counts compared to the PCR-negative group (p<0.001). The number of biopsy fragments did not affect CMV detection by immunohistochemistry.

***Conclusion:*** While evaluating endoscopic biopsies of patients with inflammatory bowel disease, CMV immunohistochemistry assessment as a reflex test may be considered by the pathologist—even in the absence of identifiable viral cytopathic effects with hematoxylin-eosin—particularly when severe histologic inflammation is present. Although the clinical significance of CMV immunohistochemistry could not be fully determined in this study, this approach may increase the likelihood of detecting CMV infection and, in the appropriate clinical context, could contribute to timely diagnosis and management.

## INTRODUCTION

Cytomegalovirus (CMV) is a double-stranded DNA virus belonging to the *Herpesviridae *family, first isolated approximately half a century ago (1). It has high global seroprevalence and can cause various clinical manifestations in both immunocompetent and immunocompromised individuals (2,3).

Following the initial description of CMV infection in a patient with ulcerative colitis (UC) (4), several studies have investigated the association between CMV infection/reactivation and inflammatory bowel diseases (IBD) (5–8). Despite conflicting evidence regarding whether CMV positivity contributes to the exacerbation of inflammation or merely represents an epiphenomenon, CMV is detected in approximately 30% of patients with IBD, with a notably higher prevalence in steroid-refractory cases (9). CMV infection has also been associated with severe colitis (10–12). Some studies have reported that CMV-positive IBD patients are more likely to require hospitalization and have an increased risk of colectomy; however, these associations have not been consistently confirmed (13–17). While certain reports suggest that antiviral therapy does not significantly alter overall outcomes, CMV-positive IBD cases that are refractory to corticosteroids may benefit from a more individualized therapeutic approach (10,18).

Nevertheless, the optimal method for detecting CMV reactivation remains unclear. Studies indicate that serum CMV PCR or tissue CMV PCR -particularly in ulcerated areas- can yield positive results during acute severe disease and may be associated with clinical outcomes (18). In addition to PCR-based testing, histological evidence of CMV in tissue, either through morphological assessment or immunohistochemistry, is critical for diagnosis and has prognostic significance in patients with IBD (11,15,16,19,20).

Given the clinical significance of CMV positivity in IBD patients, we aimed to investigate the association between positive CMV immunohistochemistry (IHC) results and clinical, endoscopic, histological and tissue CMV PCR findings in ileocolonoscopic biopsies of IBD patients, both with and without a preliminary clinical diagnosis of CMV infection. Additionally, we aimed to assess the diagnostic impact of CMV IHC utility in routine histopathologic evaluation of endoscopic biopsies of IBD patients.

## MATERIALS and METHODS

Pathology reports of ileocolonoscopic biopsies from patients clinically and endoscopically diagnosed with IBD, in whom CMV IHC was performed between 2018 and 2021, were retrospectively evaluated at a tertiary reference center. This study was approved by the Clinical Research Ethics Committee of the Istanbul University Faculty of Medicine (file number: 2021/1810).

The distinction between UC and CD was made based on endoscopic biopsies on clinical grounds. Cases in which a definitive distinction could not be established were excluded from the study. The clinical and endoscopic findings were retrieved from the hospital database. Mayo endoscopic scores of ileocolonoscopy findings were recorded. Normal endoscopic appearance was scored as ‘0’; erythema, decreased vascular pattern, mild friability were scored as ‘1’ (mild activity); marked erythema, absent vascular pattern, friability, erosions were scored as ‘2’ (moderate activity); and spontaneous bleeding, ulceration were scored as ‘3’ (severe activity) (21). The extent of disease involvement was determined based on endoscopic evaluation. The classification of involvement included ileal, ileocolonic, colonic involvement, and pancolitis.

All archived H&E-stained biopsy sections were re-examined. Biopsies were evaluated for histologic disease activity using the Simplified Geboes Score (SGS) (22). The SGS comprises five grades: Grade 0 indicates no inflammatory activity; Grade 1 is characterized by the presence of basal plasma cells; Grade 2 is defined by eosinophils or neutrophils in the lamina propria; Grade 3 denotes the presence of neutrophils in the epithelium; and Grade 4 reflects epithelial injury in the crypts and surface epithelium, as evidenced by crypt destruction, erosion, ulceration, or granulation tissue formation. The highest grade observed across the various colonic sites was used to determine the overall activity score.

In addition, biopsies were re-examined for CMV-associated viral cytopathic changes, such as nuclear inclusions, nuclear and cytoplasmic amphophilic appearance, and cellular enlargement. Tissue fragments that underwent CMV IHC were counted and measured.

### CMV immunohistochemistry

Four µm-thick tissue sections of the biopsies were incubated with the primary CMV antibody (CMV Novocastra, 1/200 dilution, 32 minutes incubation) by using an automated staining module (Ventana Medical System-Benchmark XT/ISH Staining Module, Roche, Switzerland).

The total number of CMV IHC-positive cells was counted for each case. In addition, the number of stained cells in the area with the highest staining density at a medium power (20× objective) was recorded (20).

### Statistical Analysis

All statistical analyses were conducted using the Statistical Package for the Social Sciences (SPSS) software, version 28.0 for Windows (IBM Corp., Armonk, NY, USA). The Kolmogorov–Smirnov test was used to assess the normality of the data distribution. Descriptive statistics were presented as mean±standard deviation for continuous variables, and as frequency and percentage for categorical variables. Differences in mean values between groups were analyzed using the independent samples t-test. Associations between categorical variables were evaluated using Pearson’s chi-square test. A p-value <0.05 was considered statistically significant.

## RESULTS

### Patient Characteristics, Including Demographic, Clinical and Histologic Data

A total of 191 patients underwent CMV immunohistochemistry, including 136 patients (71.2%) with UC and 55 patients (28.8%) with Crohn’s disease (CD). The mean age of the patients was 43.17 years (±15.6 SD; range: 18-76 years). Patients with CD were significantly younger than those with UC (*p=0.001*). Of the patients, 68% (n=130) were male and 32% (n=61) were female. There was no significant gender difference between the UC and CD groups (*p=0.063*).

Of the total cohort, 82 patients (42.9%) presented with symptoms such as bloody mucous stool, diarrhea, abdominal pain, and weight loss, while 109 patients (57.1%) were asymptomatic and underwent colonoscopy for surveillance. Symptomatic presentation was more frequent in UC (51.5%) compared to CD (21.8%) (*p<0.001*).

Biopsies from 13 patients (6.8%) were initial diagnostic samples, while those from 178 (93.2%) patients were obtained during follow-up or relapse.

In patients with CD, ileal (n=14, 24.5%), ileocolonic (n=26, 47.3%), and colonic (n=113, 24.5%) involvement were observed, with ileocolonic involvement being the most common. Pancolitis was noted in only one patient (1.8%) with CD. In contrast, among patients with UC, colonic involvement was observed in the majority of cases (n= 99, 72.8%), while pancolitis was present in 37 cases (27.2%) (*p<0.001*).

There was no significant difference in Mayo endoscopic scores between UC and CD (*p=0.158*); however, a significant difference was observed in the SGS (*p=0.008*). Approximately three-quarters of CD patients had histologic activity corresponding to SGS 4. In contrast, among UC patients 73 (53.7%) had SGS 4, followed by SGS 3 in 51 (37.5%).

**Figure 1:**
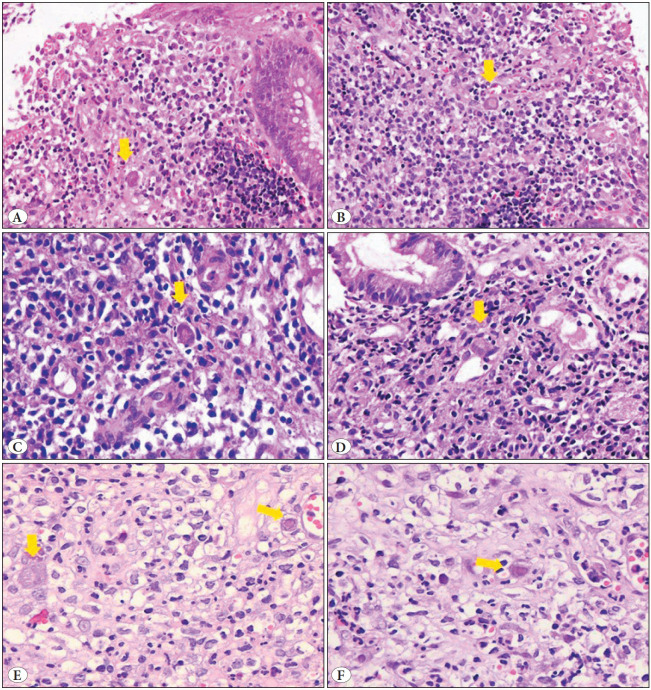
Histopathological findings and CMV-associated viral cytopathic changes on H&E. Ulceration and highly active inflammation with a SGS of 3-4 are observed in figures **(A,B)**. CMV-associated cytoplasmic and nuclear enlargement, along with amphophilic cytoplasmic staining, are observed in endothelial and stromal cells marked with arrows **(A-F)**. Intranuclear eosinophilic inclusions are obvious in figures b, c, e (right arrow), and f.

Viral cytopathic changes on H&E staining were observed in only 19 patients (9.9%), predominantly in UC (n=18, 94.7%), and only in one patient (5.3%) with CD (*p=0.017*). Histologic findings are shown in Figure 1.

The clinicopathologic features of the patients are presented in detail in Table 1.

**Table 1:** Clinicopathologic features of the patients

	**All patients (n=191)**	**UC (n=136, 71.2%)**	**CD (n=55, 28.8%)**	***p*** **value**
Age (mean±SD)	43.17±15.6	45.48±15.16	37.47±15.71	***0.001***
Gender Male Female	130 (68%) 61 (32%)	98 (75.4%) 38 (62.3%)	32 (24.6%) 23 (37.7%)	*0.063*
Extension Ileal Ileocolonic Colonic Pancolitis	14 (7.3%) 26 (13.6%) 113 (59.2%) 38 (19.9%)	0 (0%) 0 (0%) 99 (72.8%) 37 (27.2%)	14 (24.5%) 26 (47.3%) 14 (24.5%) 1 (1.8%)	***<0.001***
Biopsy time First diagnostic biopsy Follow up or relapse biopsy	13 (6.8%) 178 (93.2%)	11 (8%) 125 (92%)	2 (3.6%) 53 (96.3%)	*0.269*
Clinic presentation Symptomatic Asymptomatic	82 (42.9%) 109 (57.1%)	70 (51.5%) 66 (48.5%)	12 (21.8%) 43 (78.2%)	***<0.001***
Mayo endoscopic score 0 1 2 3	4 (2.1%) 17 (8.9%) 19 (9.9%) 151 (79.1%)	4 (3%) 14 (10.2%) 16 (11.8%) 102 (75%)	0 (0%) 3 (5.5%) 3 (5.5%) 49 (89%)	*0.158*
Simplified Geboes score 0 1 2 3 4	- 6 (3.1%) 11 (5.8%) 59 (30.9%) 115 (60.2%)	- 3 (2.2%) 9 (6.6%) 51 (37.5%) 73 (53.7%)	- 3 (5.5%) 2 (3.6%) 8 (14.5%) 42 (76.4%)	***0.008***
CMV inclusion on H&E Present Absent	19 (9.9%) 172 (90.1%)	18 (94.7%) 118 (68.6%)	1 (5.3%) 54 (31.4%)	***0.017***

**H&E:** Haematoxylin-Eosin

### CMV Immunohistochemistry Results

CMV IHC positivity was observed in 62 cases (32.4%), while 129 cases (67.6%) showed no immunoreactivity. The CMV IHC-positive patients were significantly older (mean±SD: 46.47±15.27 years) than the negative patients (mean±SD: 41.59±15.73) (*p=0.044*), with no significant difference in gender (*p=0.112*). CMV positivity was more frequent in UC (39%) compared to CD (16.4%) (*p=0.003*) and was significantly associated with symptomatic presentation (*p=0.001*).

CMV IHC positivity was detected 8 of the cases (61.5%) at initial diagnosis, while it was identified in 54 cases (30.3%) during follow-up or relapse (*p=0.02*).

A significant association was found between CMV IHC positivity and extension of disease involvement (*p<0.001*). No CMV immunoreactivity was detected in CD cases with ileal involvement, and only 11.5% (n=3) of the CD cases with ileocolonic involvement were positive. In contrast, CMV IHC positivity was observed in 34.5% of cases (n=39) with colonic involvement (34 cases with UC and 5 with CD) and in 52.6% of cases (n=20) with pancolitis (19 with UC and 1 with CD).

Although no association was found between CMV IHC positivity and the Mayo endoscopic score (*p=0.337*), a significant association was detected with the SGS. No immunoreactivity was observed in cases with SGS 1 or 2. Among CMV IHC-positive cases, 83.8% (n=52) exhibited histologic activity corresponding to SGS 4, while the remaining 16.2% (n=10) showed SGS 3 activity (*p<0.001*).

**Figure 2:**
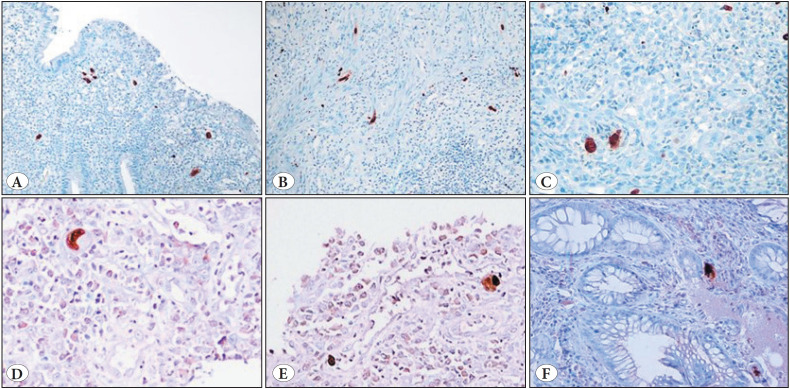
CMV IHC findings demonstrating CMV-positive cells within inflamed mucosal tissues. Variable intensities of CMV IHC immunoreaction are observed in areas of active inflammation **(A-F)**. Depending on the plane of sectioning, some positive signals appear smaller, while others appear larger. Cellular enlargement is more clearly visualized in sections passing through the central axis of the cell **(C-F)**.

**Figure 3:**
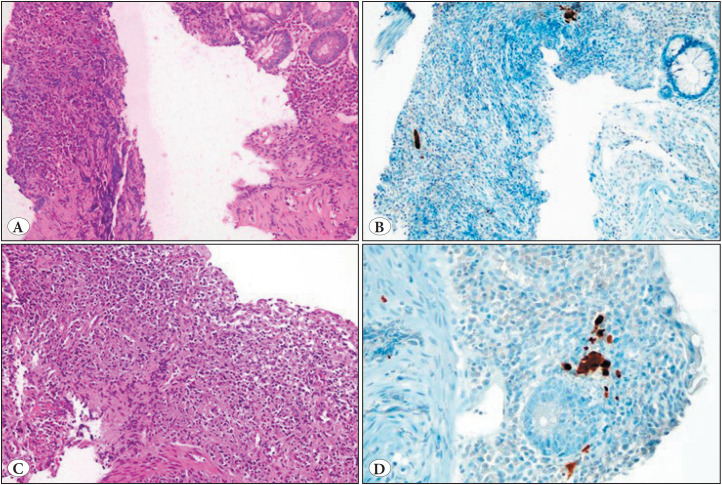
CMV IHC findings demonstrating CMV-positive cells within inflamed mucosal tissues. No visible cytopathic changes are seen in the inflamed areas on H&E staining **(A,C)**, whereas positive immunoreactivity is observed on IHC **(B,D)**. A crush artifact on the left side of figure **(A)** complicates histological evaluation.

CMV IHC positivity was significantly associated with the presence of ulceration (83.9%, *p<0.001*). Particularly, among cases with a SGS 4, 41 (66.1%) showed CMV positivity only in ulcerated tissue, 7 (11.3%) showed positivity in both ulcerated and non-ulcerated tissues, and 4 (6.4%) showed positivity exclusively in non-ulcerated tissue. The remaining 10 (16.2%) CMV-positive cases, all with a SGS 3 displayed positivity in actively inflamed tissue. CMV IHC findings are presented in Figure 2 and Figure 3.

The mean number of tissue fragments (0.2–0.3 cm in diameter colonoscopic biopsies) subjected to CMV IHC was 2.82 ± 1.72 (range: 1–14). The distribution of tissue amounts were as follows: 1 tissue in 33 cases, 2 in 65 cases, 3 in 53 cases, 4 in 16 cases, 5 in 8 cases, 6 in 7 cases, 7 in 6 cases, 8 in 2 cases, and 14 in 1 case. There was no significant difference in the number of tissues between the CMV IHC-positive and -negative groups. The CMV-positive group had a mean of 3.02±2.05 (range: 1–14) tissues, compared to 2.74±1.55 (range: 1–8) in the CMV-negative group (*p=0.297*).

The association between CMV IHC positivity and clinicopathologic features is summarised in Table 2.

**Table 2:** Association between CMV IHC positivity and clinicopathologic features

	**CMV positive** **(n=62, 32.4%)**	**CMV negative** **(n=129, 67.6%)**	***p*** **value**
Age (mean±SD)	46.47±15.27	41.59±15.73	***0.044***
Gender Male (n=130) Female (n=61)	47 (36.1%) 15 (24.6%)	83 (63.9%) 46 (75.4%)	*0.112*
Diagnosis UC (n=136) CD (n=55)	53 (39%) 9 (16.4%)	83 (61%) 46 (83.6%)	** ** ***0.003***
Symptom Present (n=82) Absent (n=109)	37 (59.7%) 25 (40.3%)	45 (34.9%) 84 (65.1%)	***0.001***
Mayo endoscopic score 0 (n=4) 1 (n=17) 2 (n=19) 3 (n=151)	0 (0%) 4 (6.4%) 8 (13%) 50 (80.6%)	4 (3.1%) 13 (10%) 11 (8.5%) 101 (78.3%)	*0.337* *0.164* (0+1vs2+3)
Simplified Geboes score 0 (n=0) 1 (n=6) 2 (n=11) 3 (n=59) 4 (n=115)	- 0 (0%) 0 (0%) 10 (16.2%) 52 (83.8%)	- 6 (4.6%) 11 (8.6%) 49 (38%) 63 (48.8%)	***<0.001*** ***0.003*** **(0+1+2vs3+4)**
Extension Ileal (n=14) Ileocolonic (n=26) Colonic (n=113) Pancolitis (n=38)	0 (0%) 3 (11.5%) 39 (34.5%) 20 (52.6%)	14 (100%) 23 (88.5%) 74 (65.5%) 18 (47.4%)	*** *** ***<0.001***
Biopsy time First diagnostic biopsy (n=13) Follow up or relapse biopsy (n=178)	8 (61.5%) 54 (30.3%)	5 (38.5%) 124 (69.7%)	*** *** ***0.020***
Ulceration on histopathology Present (n=115) Absent (n=76)	52 (83.9%) 10 (16.1%)	63 (48.8%) 66 (51.2%)	*** *** ***<0.001***
CMV inclusion on H&E Present (n=19) Absent (n=172)	19 (30.6%) 43 (69.4%)	0 (0%) 129 (100%)	*** *** ***<0.001***
Tissue amount in IHC slide (no. of core bx) (mean±SD) (median)	3.02±2.05 (1-14) 3	2.74±1.55 (1-8) 2	* * *0.297*
Tissue CMV PCR (n=101) Positive (n=61) Negative (n=40)	35 (57.4%) 6 (15%)	26 (42.6%) 34 (85%)	* * ***<0.001***

### Cases with and without a Preliminary Clinical Diagnosis of CMV Infection

Among the 191 patients, 36 (18.8%) had a preliminary clinical diagnosis of CMV infection of which 22 (61.1%) were symptomatic. In contrast, among 155 patients (81.2%) without a preliminary clinical diagnosis of CMV infection, 60 patients (38.7%) were symptomatic (*p=0.014*). There was no significant association between the underlying disease (UC or CD) and whether there was preliminary clinical diagnosis of CMV infection (*p=0.169*).

Among CMV IHC-positive cases, only 25.8% had a preliminary clinical diagnosis of CMV infection, while the remaining 74.2% were identified by the pathologist without any prior clinical suspicion of CMV infection. Association between preliminary clinical diagnosis of CMV infection and CMV IHC results are summarized in Table 3.

**Table 3:** Association between preliminary clinical diagnosis of CMV infection and CMV IHC results

**Performing CMV immunohistochemistry**			
	**With preliminary clinical diagnosis (n=36, 18.8%)**	**Without preliminary clinical diagnosis (n=155, 81.2%)**	***p*** **value**
Diagnosis UC (n=136) CD (n=55)	29 (21.3%) 7 (12.7%)	107 (78.7%) 48 (87.3%)	*0.169*
Clinic presentation Symptomatic (n=82) Asymptomatic (n=109)	22 (61.1%) 14 (38.9%)	60 (38.7%) 95 (61.3%)	***0.014***
Mayo endoscopic score 0 (n=4) 1 (n=17) 2 (n=19) 3 (n=151)	0 (0%) 1 (2.8%) 2 (5.6%) 33 (91.7%)	4 (2.6%) 16 (10.3%) 17 (11%) 118 (76.1%)	*0.208* * * *0.080* (0+1vs2+3)
Simplified Geboes score 0 (n=0) 1 (n=6) 2 (n=11) 3 (n=59) 4 (n=115)	- 0 (0%) 1 (2.8%) 9 (25%) 26 (72.2%)	- 6 (3.9%) 10 (6.5%) 50 (32.3%) 89 (57.4%)	*0.303* * * *0.152* (0+1+2vs3+4)
CMV inclusion on H&E Present (n=19) Absent (n=172)	6 (16.7%) 30 (83.3%)	13 (8.4%) 142 (91.6%)	*0.135*
CMV immunohistochemistry Positive (n=62) Negative (n=129)	16 (25.8%) 20 (15.5%)	46 (74.2%) 109 (84.5%)	*0.088*
Total number of IHC-positive cells per slide (mean±SD)	6.5±4.3	8.9±12.8	* * *0.27*
Number of IHC-positive cells at 20× magnification (mean±SD)	2.8±1.7	4.6±5.7	* * *0.06*

### Quantification of Cells Exhibiting Viral Cytopathic Changes and CMV IHC Positivity

Among the 62 CMV IHC-positive cases (32.4%), 19 (30.6%) had viral cytopathic changes on H&E, while 43 (69.4%) showed no viral cytopathic changes. The detection of viral cytopathic changes on H&E was significantly correlated with CMV IHC positivity (*p<0.001*). The mean number of cells showing cytopathic changes on H&E was 0.5 ± 0.97 (range: 0–4). The mean total number of CMV IHC-positive cells per slide was 8.34 ± 11.28 (range: 1–80), and the mean number of positive cells per field at 20× magnification was 4.19 ± 5.07 (range: 1–30).

CMV IHC-positive cell counts, and cell types were analyzed separately for UC and CD. Among IHC-positive cases, the mean number of CMV-positive cells was 3.04 ± 5.63 in UC and 1.82 ± 10.82 in CD (*p=0.31*). Similarly, the mean number of positive cells per 20× field was 4.06 ± 4.03 in UC and 4.89 ± 9.51 in CD (*p=0.161*).

CMV IHC positivity was observed in endothelial cells and stromal cells. The distribution of positive cell types was as follows: 31 cases (52%) showed positivity in both endothelial and stromal cells; 22 cases (35.4%) showed positivity only in endothelial cells; 9 cases (14.6%) showed positivity only in stromal cells. There was no significant difference in the distribution of IHC-positive cell types between UC and CD (p=0.11). Details are presented in Table 4.

**Table 4:** Quantification of positive cells in CMV IHC by disease type

	**UC** **CMV IHC positive** **n=53 (39%)**	**UC** **CMV IHC negative** **n=83 (61%)**	***p*** **value**	**CD** **CMV IHC positive** **n=9 (16.4%)**	**CD** **CMV IHC** **negative** **n=46 (83.6%)**	***p*** **value**
Simplified Geboes score 0 (n=0) 1 (n=6) 2 (n=11) 3 (n=59) 4 (n=115)	- 0 0 10 (19.6%) 43 (59%)	- 3 (100%) 9 (100%) 41 (80.4%) 30 (42%)	* * ***<0.001***	- 0 (0%) 0 (0%) 0 (0%) 9 (21.4%)	- 3 (100%) 2 (100%) 8 (100%) 33 (78.6%)	* * *0.343*
Tissue amount in IHC slide (no. of core bx) (mean±SD)	3.02±2.08	2.64±1.66	Not significant	3.00±2.00	2.91±1.35	Not significant
CMV inclusion on H&E Present (n=19) Absent (n=172)	18 (100%) 35 (30%)	0 (0%) 83 (70%)	***<0.001***	1 (100%) 8 (14.8%)	0 (0%) 46 (85.2%)	***0.024***
Total number of IHC-positive cells per slide (mean±SD)	3.04±5.63	-	N/A	1.82±10.82	-	N/A (*p=0.31* UC vs CD)
Number of IHC-positive cells at 20× magnification (mean±SD)	4.06±4.03	-	N/A	4.89±9.51	-	N/A (*p=0.16 *UC vs CD)
IHC positive cell type E (n=22) S (n=9) E+S (n=31)	18 (34%) 6 (11.3%) 29 (54.7%)	-	N/A	4 (44.4%) 3 (33.3%) 2 (22.2%)	-	N/A (*p=0.11 *UC vs CD)

**E:** Endothelial cell, **S:** Stromal cell, **H&E: **Haematoxylin-Eosin

No significant difference was found in the total number of CMV-positive cells with or without clinically mentioned CMV suspicion (6.5±4.3 vs 8.9±12.8, *p=0.27*). Although there was higher mean number of positive cells per 20× field without a preliminary clinical diagnosis, this difference did not reach statistical significance (2.8±1.7 vs 4.6±5.7, *p=0.06*).

### Correlation between CMV IHC results with tissue CMV PCR results

The data of tissue CMV PCR results was obtained in 101 (52.9%) cases, of whom 61 (60.4%) were positive. Tissue CMV PCR results showed a statistically significant correlation with CMV IHC findings (*p<0.001*).

We conducted a statistical analysis within the cohort of patients who underwent tissue CMV PCR testing (n=101), focusing on the number of IHC-positive cells and the presence of viral cytopathic changes on H&E staining.

Our analysis revealed that the PCR-positive group had significantly higher IHC-positive cell counts compared to the PCR-negative group. The mean total number of positive-stained cells per slide was 6.21±11.47 in the PCR-positive group versus 0.45±1.45 in the PCR-negative group *(p<0.001)*. Similarly, the number of IHC-positive cells at 20× magnification was significantly higher in the PCR-positive group (2.93±4.7 vs. 0.3±1.02, *p<0.01*). Moreover, CMV inclusions on H&E staining were observed exclusively in the PCR-positive group *(p<0.001)*.

Among the PCR-positive cases, 35 (57.4%) were also CMV IHC-positive, while 26 (42.6%) were CMV IHC-negative. Notably, six cases (14.6%) were CMV IHC-positive but PCR-negative. In the six cases that were CMV IHC-positive but tissue PCR-negative, the number of IHC-positive cells ranged from 1 to 8 (one cell in two cases, two cells in two cases, four cells in one case, and eight cells in one case), with a mean of 3 ± 2.68. In contrast, among the 35 cases that were positive for both CMV IHC and tissue PCR, the number of IHC-positive cells ranged from 2 to 80, with a mean of 10.83 ± 13.44. Although a higher number of positive cells was observed in patients positive for both IHC and PCR, the difference was not statistically significant *(p>0.05)*. This lack of significance may be due to the small sample size of the IHC-positive/PCR-negative group. In addition, no significant clinical differences were observed between IHC-positive/PCR-negative and IHC-positive/PCR-positive patients in terms of diagnosis, gender, clinical presentation at the time of biopsy, disease severity (as assessed by the simplified Geboes score), or Mayo endoscopic score (*p>0.05* for all). However, the presence of viral cytopathic changes on H&E staining differed significantly between the IHC-positive/PCR-positive and IHC-positive/PCR-negative groups. None of the six IHC-positive/PCR-negative cases showed viral cytopathic changes on H&E. In contrast, 48.6% of the IHC-positive/PCR-positive cases (n=17) exhibited such changes (p=0.033). In the remaining IHC-positive/PCR-positive cases (n=18, 51.4%), no cytopathic effects were identified on H&E.

The comparison of CMV IHC and Tissue CMV PCR results are given in Table 5.

**Table 5:** The comparison of CMV IHC and tissue CMV PCR results

	**Tissue CMV PCR positive** **n=61**	**Tissue CMV PCR negative** **n=40**	***p*** **value**
CMV IHC Positive (n=41) Negative (n=60)	35 26	6 34	***<0.001***
Total number of IHC-positive cells per slide (mean±SD) Range (median)	6.21±11.47 0-80 (3)	0.45±1.45 0-8 (0)	*** *** ***<0.001***
Number of IHC-positive cells at 20× magnification (mean±SD) Range (median)	2.93±4.7 0-30 (1)	0.3±1.02 0-6 (0)	*** *** ***<0.001***
CMV inclusion on H&E Present (n=17) Absent (n=84)	17 44	0 40	*** *** ***<0.001***
Number of inclusions on H&E (mean±SD) Range (median)	0.48±0.99 0-4 (0)	0±0 0	** ** ***<0.001***

## DISCUSSION

Given the potential prognostic implications of CMV positivity in IBD, identification of CMV-positive cases is crucial. The optimal method for detecting CMV reactivation remains unclear. The most recommended method for detecting CMV infection is histological or immunohistochemical evaluation rather than PCR-based testing (23,24).

We retrospectively evaluated the relationship between CMV IHC results and clinical presentation, endoscopic findings, and histologic features in IBD patients. The quantification of cells exhibiting viral cytopathic changes and positively stained CMV IHC were also examined in detail.

CMV IHC positivity was more frequently observed in patients with UC (39%) than in those with CD (16.4%). In this retrospective study, most CMV IHC-positive cases (74.2%) had no preliminary clinical diagnosis of CMV infection. Whereas CMV IHC positivity was associated with symptomatic presentation, 45.1% of the symptomatic cases were CMV IHC positive and only 26.8% of these had a preliminary clinical diagnosis of CMV infection. Previous studies have reported an association between CMV positivity and higher Mayo endoscopic scores or the presence of endoscopic ulcers (14,17,25). No association was found in this study between the Mayo endoscopic score and CMV IHC positivity. On the other hand, a high SGS was significantly associated with CMV positivity. Approximately 91% of the cases had a high SGS (score 3 or 4), and CMV IHC positivity was observed in 35.6% of these cases.

CMV-related cytopathic effects were identified on H&E slides in only 19 cases (9.9%), whereas CMV IHC was positive in 62 cases (32.4%). This discrepancy may be explained by the difficulty in detecting CMV-related cytopathic changes on H&E-stained slides. Although interpretation is more straightforward when characteristic eosinophilic nuclear inclusions-known as “owl’s eye” appearances-are present, several factors can hinder accurate assessment. These factors include technical variables such as section thickness and staining quality, clinical limitations such as inadequate tissue sampling, and the experience level of the interpreting pathologist. It seems that a combined evaluation using H&E staining alongside IHC offers a more reliable approach for diagnosing CMV infection, particularly in tissue-based assessments (18,23,24).

In our study, an average of 8.34 ± 11.28 CMV-positive cells per biopsy specimen (range:1-80) and 4.19 ± 5.07 CMV-positive cells per field at 20× magnification (range:1-30) were detected. However, due to limited clinical data, the association between CMV density, disease course, and treatment response could not be evaluated in this study. Several studies have suggested that not merely the presence of CMV positivity on IHC, but rather the density of CMV-positive cells, is associated with prognosis, treatment response, and the need for colectomy (11,15,20,26). The presence of more than five CMV-positive cells has been linked to an increased risk of colectomy in some reports, whereas others have identified similar risk associations with as few as two positive cells (15,26). Additionally, one study reported that more than ten CMV-positive cells in a biopsy specimen was correlated with steroid resistance and acute clinical deterioration (11). These studies employed varying cut-off values for CMV-positive cell counts; while some considered the total number of positive cells per biopsy, others were focused on the most intense areas at medium power magnification (×20). Further prospective studies are needed to elucidate these potential correlations.

No standardized or universally accepted minimum number of tissue samples for reliable CMV detection via IHC has been established in the literature (24). A study has demonstrated that obtaining a minimum number of 11 colonic biopsies from inflamed tissue in patients with UC, and 16 in those with CD, is required to achieve adequate diagnostic sensitivity for CMV detection (27). In our study, there was no statistically significant difference in the mean number of tissue samples between the CMV IHC-positive and CMV IHC-negative groups. The mean and median numbers of tissue samples in the IHC positive group was only three. As also observed in our study, CMV positivity is more likely to be detected in ulcerated tissues; therefore, obtaining targeted biopsies from the ulcerated tissues may enhance the likelihood of CMV detection even with a reduced number of tissue samples. According to our results, suggesting that obtaining at least three samples from the actively inflamed areas may be beneficial for detecting CMV. Considering that CMV may exhibit a patchy distribution in biopsies, increasing the number of tissue samples could enhance the likelihood of detection.

Although the detection of CMV-related viral cytopathic effects via H&E and/or IHC is widely accepted as a practical diagnostic standard, some studies have suggested that tissue CMV PCR may be more reliable and recommended that CMV IHC should not be used alone but rather in combination with molecular tests (28,29). In those studies, the rates of IHC positivity among CMV PCR-positive cases were reported as 21.7% and 35.1%. In contrast, the present study demonstrated a higher IHC positivity rate of 57.4% among PCR-positive cases. Additionally, we observed a statistically significant correlation between tissue CMV PCR results and CMV IHC findings. We also found that a higher number of CMV IHC-positive cells and the presence of viral cytopathic changes on H&E staining were significantly associated with tissue PCR positivity. Notably, six cases in our cohort were CMV IHC-positive but PCR-negative, while 26 cases were CMV IHC-negative yet PCR-positive. No significant clinical differences were observed between IHC-positive/PCR-negative and IHC-positive/PCR-positive patients, except that viral cytopathic changes on H&E were present only in the IHC-positive/PCR-positive group. Previous studies have linked tissue CMV PCR positivity—particularly in the presence of high viral loads—with resistance to steroid or other immunosuppressive therapies (18). Although viral load data were not available in our study, it is reasonable to hypothesize that discordant IHC/PCR results may reflect cases with low tissue viral burden.

While the relative diagnostic performance of CMV IHC versus PCR remains debated, our findings suggest that the presence of viral cytopathic effects on H&E may serve as a potential predictor of tissue PCR positivity. However, detecting such changes can be challenging due to their subtle and sometimes nonspecific appearance. In this context, IHC serves as a valuable diagnostic adjunct -particularly in cases where low viral loads might lead to false-negative PCR results. IHC not only enhances diagnostic confidence but also provides important morphological context by highlighting infected cells within the tissue. In such instances, the direct histological detection of viral cytopathic effects or IHC positivity may offer more clinically relevant evidence of active infection than the mere presence of viral DNA detected by PCR (9).

The central question in this context is: What is the clinical significance of CMV presence in the tissue? As previously mentioned in the literature, it remains debated whether CMV plays a pathogenic role contributing to disease activation or merely represents an “innocent bystander” (9). While differing opinions exist, several studies have suggested that in terms of prognosis, treatment response, and the need for colectomy, the density of CMV IHC-positive cells -rather than mere positivity- may be a more relevant factor for decision making (10,11,15,20,26) as discussed above. Given the current lack of consensus regarding both cut-off thresholds and quantification methodology, routine reporting of the exact number of IHC-positive cells is not universally recommended. Nonetheless, pathologists may consider documenting CMV density, particularly in cases with high histologic activity, as this information could offer additional prognostic value when interpreted in the appropriate clinical context. A notable finding in this retrospective analysis of CMV IHC-positive cases was that the majority (74.2%) were identified based on the pathologist’s suspicion rather than a prior clinical suspicion. Considering that adequate clinical information and a suspicion of CMV infection in the preliminary diagnosis were not provided for every case with high Mayo endoscopic scores or a symptomatic presentation, this raises an important question: In which patient groups should pathologists suspect CMV infection and pursue further investigation? Based on our findings, and considering the established association between CMV infection and severe or steroid-refractory disease (9–11), as well as the clinical significance of isolated CMV positivity detected by IHC (30), we propose that in cases with high histological activity, specifically those with an SGS of 3 or 4, pathologists should assess for CMV infection. Moreover, we recommend that CMV presence be investigated not solely through routine histological evaluation, but also by employing IHC staining as a reflex test particularly in patients with UC may offer valuable insights for guiding clinical management, even when CMV infection is not clinically suspected. Treatment decisions should incorporate clinical context, viral load, and biopsy findings.

This study has some limitations. First, it is a retrospective study, and the cases in which CMV IHC was performed were selected from our routine pathology reports. Another limitation is the inability to evaluate the clinical implications such as data regarding the proportion of patients who received antiviral therapy, the dosage, treatment duration, and clinical outcomes as well as viral load were insufficient and thus could not be analyzed. In addition, serum CMV IgM results were not available for the patients included in this study, preventing correlation with serologic findings.

## CONCLUSION

In summary, our findings suggest that integrating histopathological, immunohistochemical, and molecular approaches may improve the detection of CMV infection in patients with IBD. The observed association between CMV IHC positivity, cytopathic changes on H&E, and higher histological activity supports the potential relevance of tissue-based evaluation. While the clinical significance of CMV IHC could not be fully determined in this study, our results indicate that CMV IHC assessment as a reflex test may be considered—particularly in UC patients with severe histologic inflammation (SGS ≥3)—as it could contribute to timely diagnosis and inform patient management in the appropriate clinical context.

## Ethical Approval

This study was approved by the Clinical Research Ethics Committee of the Istanbul University Faculty of Medicine (file number: 2021/1810).

## Conflict of Interest

There are no conflicts of interest.

## Funding

No funding.
